# A repeated measures, randomised cross-over trial, comparing the acute exercise response between passive and active sitting in critically ill patients

**DOI:** 10.1186/1471-2253-15-1

**Published:** 2015-01-13

**Authors:** Nikki Collings, Rebecca Cusack

**Affiliations:** Department of Physiotherapy, University Hospital Southampton NHS Foundation Trust, Tremona Road, SO16 6YD Southampton, UK; Anaesthesia and Critical Care Research Unit, University Hospital Southampton NHS Foundation Trust, Tremona Road, SO16 6YD Southampton, UK; Integrative Physiology and Critical Illness Group, Clinical and Experimental Sciences, University of Southampton, University Hospital Southampton, Tremona Road, SO16 6YD Southampton, UK; Southampton NIHR Respiratory Biomedical Research Unit, University Hospital Southampton, Tremona Road, SO16 6YD Southampton, UK

**Keywords:** Critical illness, Early ambulation, Exercise, Oxygen consumption, Physiotherapy, Rehabilitation

## Abstract

**Background:**

Early mobilisation of critically ill patients is safe and beneficial, but the metabolic cost of exercise remains unquantified. This study compared the acute exercise response in critically ill participants during passive and active sitting.

**Method:**

We conducted a prospective, randomised, cross-over study, in ventilated patients receiving rehabilitative physiotherapy. Ten participants completed a passive chair transfer, or a sit on the edge of the bed, followed by the alternate exercise activity on the consecutive day. The primary outcome measure was oxygen consumption.

**Results:**

In comparison to resting supine, a passive chair transfer elicited no change in oxygen consumption, carbon dioxide production or minute ventilation; but mean arterial pressure (91.86 mmHg (95% CI 84.61 to 99.10) to 101.23 mmHg (95% CI 93.35 to 109.11) (p = 0.002)) and heart rate (89.13 bpm (95% CI 77.14 to 101.13) to 97.21 bpm (95% CI 81.22 to 113.20) (p = 0.008)) increased. Sitting on the edge of the bed resulted in significant increases in oxygen consumption (262.33 ml/min (95% CI 201.97 to 322.70) to 353.02 ml/min (95% CI 303.50 to 402.55), p = 0.002), carbon dioxide production (171.93 ml/min (95% CI 131.87 to 211.98) to 206.23 ml/min (95% CI 151.03 to 261.43), p = 0.026), minute ventilation (9.97 l/min (95% CI 7.30 to 12.65) to 12.82 l/min (95% CI 10.29 to 15.36), p < 0.001), mean arterial pressure (86.81 mmHg (95% CI 77.48 to 96.14) to 95.59 mmHg (95% CI 88.62 to 102.56), p = 0.034) and heart rate (87.60 bpm (95% CI 73.64 to 101.56) to 94.91 bpm (95% CI 79.57 to 110.25), p = 0.007). When comparing the 2 activities, sitting on the edge of the bed elicited a significantly larger increase in oxygen consumption (90.69 ml/min (95% CI 44.04 to 137.34) vs 14.43 ml/min (95% CI -27.28 to 56.14), p = 0.007) and minute ventilation (2.85 l/min (95% CI 1.70 to 3.99) vs 0.74 l/min (95% CI -0.92 to 1.56), p = 0.012).

**Conclusion:**

Sitting on the edge of the bed is a more metabolically demanding activity than a passive chair transfer in critically ill patients.

## Background

Prolonged critical illness leaves survivors with long-term morbidity [1, 2], and increased associated healthcare costs [3]. Muscle weakness and fatigue are reported as the main contributors to long-term poor functional outcomes [4, 5].

Rehabilitation of the critically ill has been established as safe and feasible [6]. Emerging evidence for early mobilisation demonstrates reduction in the number of ventilator days and hospital length of stay [7, 8], as well as improving functional outcomes at hospital discharge [9–11]. Early mobilisation within this patient cohort involves a combination of passive exercise including positioning, joint range of movement, and hoist transfer to chair; and more active tasks including sitting on the edge of the bed (SOEOB), step transfer to a chair and ambulation [12–14].

The acute response to exercise in the critically ill is often quantified by measuring changes in haemodynamic and respiratory parameters [13, 15, 16]. There is little evidence examining the metabolic cost of exercise in the early phase post critical illness. There has been no study directly comparing passive and active mobilisation in this population.

The aim of this study was to quantify and compare the acute physiological response of critically ill patients during a passive chair transfer (PCT), or a SOEOB. Primary outcome measures included oxygen consumption (VO_2_) and carbon dioxide production (VCO_2_) before, during and after the exercise activity. Secondary outcome measures included minute ventilation (MV), mean arterial pressure (MAP) and heart rate (HR).

## Methods

Ethical approval was granted by the Sheffield Hallam University Ethics Committee and the South Central Strategic Health Authority Ethics Committee. Written consent was obtained from all participants and where the patient was unable to provide this, witnessed verbal consent was obtained. No proxy caregivers were used to provide consent on behalf of the patients.

This study was a prospective, repeated measures cross-over design, conducted in a 22-bed general intensive care unit in Southampton, United Kingdom. Patients were included if they were intubated and ventilated for 4 or more days, able to mobilise 10 metres or more prior to admission, with or without a walking aid and were haemodynamically stable. Haemodynamic stability was defined as a normal electrocardiogram (ECG) and resting HR of less than 50% of age predicted maximal HR as calculated by 220 – age [17], a systolic blood pressure (SBP) between 90 and 170 mmHg [9], and the absence of inotropes. Data regarding other continuous medications such as vasoactive or sedatives were not recorded. Patients were excluded if they had an pulse oximetry (S_p_O_2_) less than 90%, a P_a_O_2_/FiO_2_ ratio less than 18, or required a positive end-expiratory pressure (PEEP) over 10 cm H_2_O; a body temperature over 38°C, a haemoglobin (Hb) less than 7 g/dL, had a confirmed deep vein thrombosis (DVT) or pulmonary embolism (PE); were unresponsive to voice, or had any neurological, orthopaedic or surgical wound contraindicating mobilisation [17]. Patients were also required to be assessed as appropriate to begin rehabilitation by the treating physiotherapist. In order to reflect clinical practice, this relied upon individual clinical reasoning of the physiotherapist; there were no specific criteria.

### Interventions

All subjects were randomly allocated to a treatment sequence using a computer-generated random numbers table [18]. Intervention arm A involved the participant completing a PCT on day 1. This involved transferring the participant to a lateral transfer chair using a pat slide, and then moving them into an upright seating position. On day 2, SOEOB was undertaken; this involved the participant being assisted from lying to upright sitting, supported by the treating physiotherapist, before being returned to lying on the bed. In intervention arm B, SOEOB was performed on day 1, with a PCT on day 2. The washout period between the 2 interventions was a minimum of 12 hours, but could extend up to 48 hours if the participant’s condition was unstable and rehabilitation on the consecutive day was clinically not appropriate. The treating physiotherapist assisted the participant with the allocated exercise activity, while being observed by the researcher.

### Outcome measures

The primary outcome measures were VO_2_ and VCO_2_ as measured by the CCOX module via the Engström Elvira ventilator (EVV). The paramagnetic O_2_ sensor and the infrared CO_2_ sensor measure the breath-by-breath inspired and expired fractions of the respective gases. The flow and volume generated by each breath is simultaneously measured by the D-lite flow sensor [19]. Secondary outcome measures included MV calculated by multiplying respiratory rate (RR) and tidal volume (TV) as recorded by the EVV, mean arterial pressure (MAP) measured via the arterial line, if present, or a non-invasive BP cuff, and HR as recorded by ECG trace.

Data for each of the 5 parameters were collected throughout 4 time periods; baseline, preparation, activity and recovery phases. Patients were left undisturbed in bed for 20 minutes prior to the commencement of data collection. Baseline measurements involved recording 3 sets of parameters at 5-minute intervals over a 10-minute period prior to any activity occurring. The preparation period consisted of minute-by-minute readings of parameters while the patient was prepared for the activity, often involving rolling of the patient; this period was variable in length, but lasted a maximum of 10 minutes. The activity phase began when the patient achieved the upright sitting position, whether completing PCT or SOEOB, and parameters were recorded minute-by-minute for 10 minutes. After completion of the activity, the patient rested for 20 minutes. Following this time, 3 sets of parameters were recorded at 5-minute intervals over a 10-minute period, called the recovery phase.

### Statistical analysis

A sample size of 10 participants was required to provide a power of 80%, at a significance level of 0.05, to detect a mean difference in VO_2_ of 115 ml/min and a standard deviation (SD) of 78 ml/min [20]. Patients were identified by the treating physiotherapist for potential suitability for the study; those meeting the inclusion criteria and able to provide consent were entered into the study and randomised.

All statistical analyses were performed using SPSS for Windows (version 17.0, SPSS, Chicago, IL, USA). Descriptive statistics included means and 95% confidence intervals for continuous measures and counts and percentages for categorical measures. All statistical tests were two-sided and significance was determined at the 0.05 probability level. The Shapiro-Wilk test assessed normality of the data sets. Normally distributed data were analysed using a paired t-test; where data was not normally distributed, a Wilcoxon matched pairs test was used for analysis.

Data collected during preparation, activity and recovery phases were compared to that at baseline, for each condition of PCT and SOEOB. Change scores between baseline and activity phases were then compared between conditions of PCT and SOEOB.

## Results

**Figure 1:**
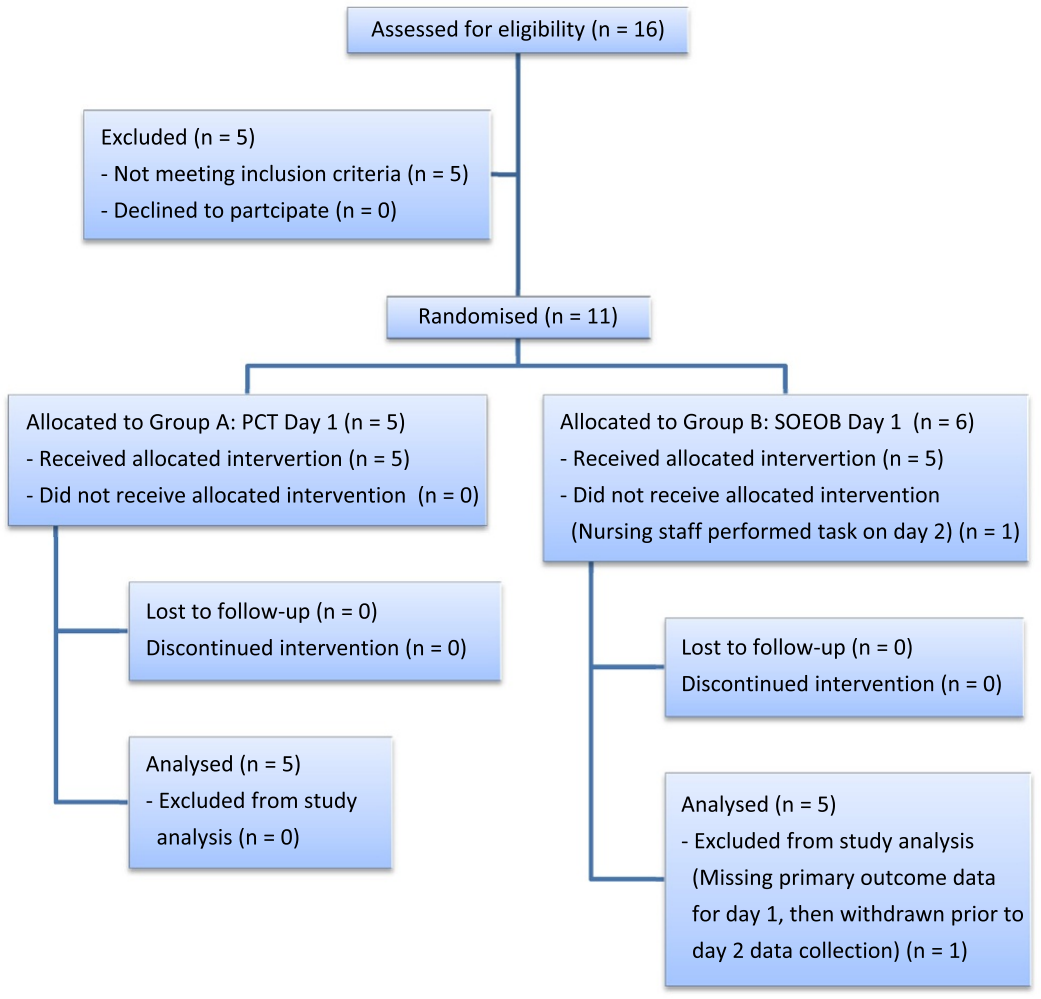
**Participant flow diagram.**

In the current study, a convenience sample of 16 patients was assessed for eligibility between over an 18 month period ending in March 2013. Five did not meet the inclusion criteria, thus 11 patients were randomised; 5 into group A and 6 into group B. One participant in group B was withdrawn from the study as no primary outcome data were collected due to equipment error during SOEOB on day 1, and human error during PCT on day 2 (see Figure 1).

Demographic data are shown in Table 1. All patients were ventilated via an assist-mode and had a PEEP of 5cmH_2_O. Patients randomised to study arm B were intubated, on average, for 7 (95% CI 1.21 to 12.79) more days at the point of recruitment, with a lower P_a_O_2_/FiO_2_ ratio: 29.7 (95% CI 19.61 to 39.79) in study arm B, versus 33.2 (95% CI 31.34 to 35.06) in study arm A; and higher resting VO_2_: 281.8 (95% CI 192.66 to 370.94) ml/min in group B, versus 250.8 (95% CI 238.97 to 262.63) ml/min in group A. Group B also had higher APACHE II scores at 20.6 (95% CI 15.04 to 26.16) vs 16.8 (95% CI 12.86 to 20.74) in group A.

**Table 1:** **Participant demographic baseline data**

Parameter	Study arm A (n = 5)	Study arm B (n = 5)
Diagnosis (no. and %)		
COPD	0	1 (20%)
Gastrectomy	0	1 (20%)
Pancreatitis	1 (20%)	2 (40%)
Polytrauma	1 (20%)	0
Pneumonia	2 (40%)	1 (20%)
Tonsillitis	1 (20%)	0
APACHE II scores (mean (*95% confidence interval*))	16.8 *(15.04 to 26.16)*	20.6 *(12.86 to 20.74)*
Age in yrs (mean ± SD) (mean (*95% confidence interval*))	61.4 *(44.68 to 78.12)*	59.2 *(31.43 to 86.97)*
Gender – male (no. and %)	4 (80%)	2 (40%)
Body mass index (mean ± SD) (mean (*95% confidence interval*))	23.2 *(18.74 to 27.58)*	25.9 *(12.45 to 39.31)*
Days intubated at recruitment (mean ± SD) (mean (*95% confidence interval*))	11.8 *(-4.68 to 28.28)*	18.8 *(5.77 to 31.83)*
Baseline P_a_O_2_/FiO_2_ ratio (mean (*95% confidence interval*))	33.2 *(31.34 to 35.06)*	29.7 *(19.61 to 39.79)*
Baseline VO_2_ in ml/min (mean (*95% confidence interval*))	250.8 *(238.97 to 262.63)*	281.8 *(192.66 to 370.94)*

**Figure 2:**
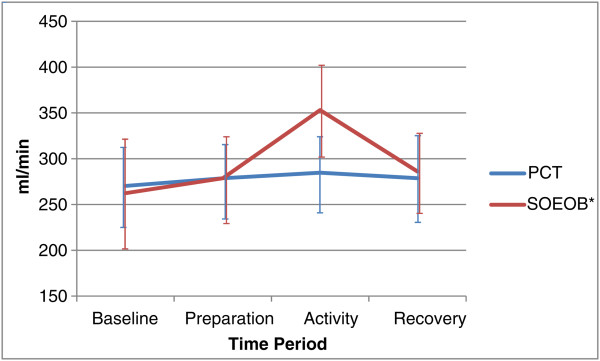
**Oxygen consumption (VO**_**2**_ **) during conditions of PCT and SOEOB.** Legend: Mean ± SD shown for PCT and SOEOB during each time period. *Significant difference between baseline and activity (p = 0.002).

**Figure 3:**
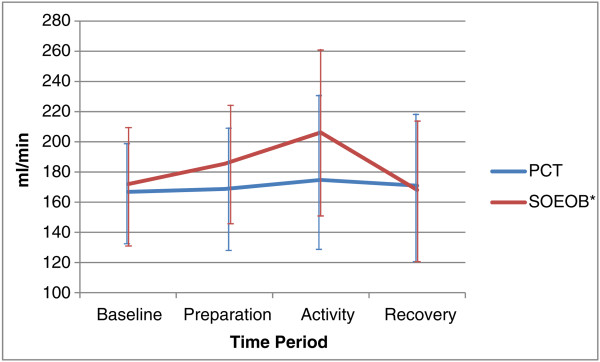
**Carbon dioxide consumption (VCO**_**2**_ **) during conditions of PCT and SOEOB.** Legend: Mean ± SD shown for PCT and SOEOB during each time period. *Significant difference between baseline and activity (p = 0.026).

**Figure 4:**
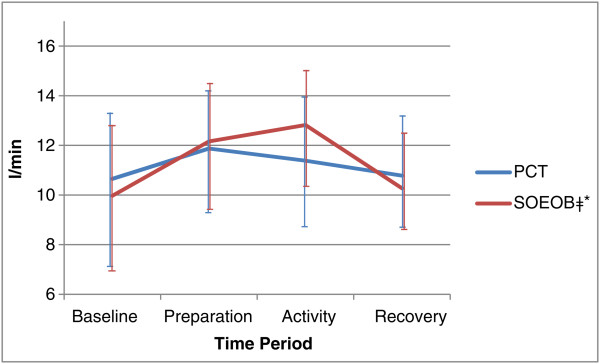
**Minute ventilation (MV) during conditions of PCT and SOEOB.** Legend: Mean ± SD shown for PCT and SOEOB during each time period. ‡Significant difference between baseline and preparation (p = 0.009). *Significant difference between baseline and activity (p < 0.001).

**Figure 5:**
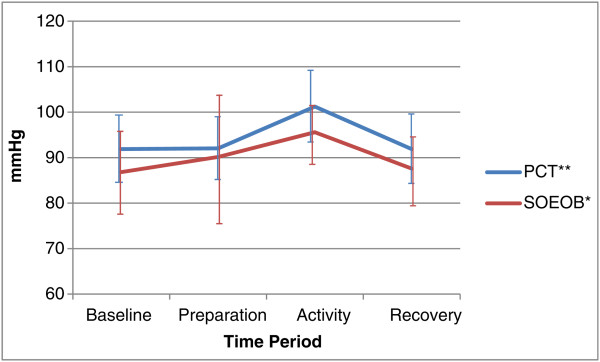
**Mean arterial pressure (MAP) during conditions of PCT and SOEOB.** Legend: Mean ± SD shown for PCT and SOEOB during each time period. *Significant difference between baseline and activity (p = 0.002).

**Figure 6:**
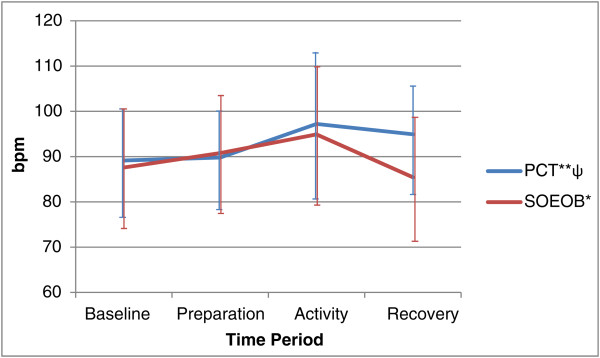
**Heart rate (HR) during conditions of PCT and SOEOB.** Legend: Mean ± SD shown for PCT and SOEOB during each time period. *Significant difference between baseline and activity (p = 0.002).

VO_2_ and VCO_2_ increased during both PCT and SOEOB (see Figures 2 and 3). During PCT, VO_2_ and VCO_2_ were not significantly different, increasing from 270.27 ml/min (95% CI 224.78 to 315.75) to 284.69 ml/min (95% CI 241.32 to 328.06), p = 0.454, and 166.80 ml/min (95% CI 134.18 to 199.42) to 174.73 ml/min (95% CI 126.47 to 222.98), p = 0.466, respectively. Significant changes were seen during SOEOB, VO_2_ increasing from 262.33 ml/min (95% CI 201.97 to 322.70) to 353.02 ml/min (95% CI 303.50 to 402.55), p = 0.002 and VCO_2_ increasing from 171.93 ml/min (95% CI 131.87 to 211.98) to 206.23 ml/min (95% CI 151.03 to 261.43), p = 0.026. During PCT, greater changes in MV were seen between the baseline and preparation phases, (10.65 l/min (95% CI 7.62 to 13.68) to 11.87 l/min (95% CI 9.43 to 14.31), p = 0.141)), than between the baseline and activity phases (11.38 l/min (95% CI 8.76 to 14.00), p = 0.076), but none of these changes were statistically significant. During SOEOB, MV rose significantly, from baseline at 9.97 l/min (95% CI 7.30 to 12.65), to the preparation phase at 12.16 l/min (95% CI 9.74 to 14.59) (p = 0.009) and baseline to the activity phase at 12.82 l/min (95% CI 10.29 to 15.36) (p < 0.001) (see Figure 4). There were significant increases in MAP from baseline to the activity phases of both PCT, from 91.86 mmHg (95% CI 84.61 to 99.10) to 101.23 mmHg (95% CI 93.35 to 109.11) (p = 0.002), and SOEOB, from 86.81 mmHg (95% CI 77.48 to 96.14) to 95.59 mmHg (95% CI 88.62 to 102.56) (p = 0.034). HR mirrored this trend, increasing significantly form baseline to the activity phase during PCT, from 89.13 bpm (95% CI 77.14 to 101.13) to 97.21 bpm (95% CI 81.22 to 113.20) (p = 0.008), and during SOEOB, from 87.60 bpm (95% CI 73.64 to 101.56) to 94.91 bpm (95% CI 79.57 to 110.25) (p = 0.007) (see Figures 5 and 6). Under the condition of PCT, HR remained elevated from baseline following the recovery phase at 94.93 bpm (95% CI 82.89 to 106.98) (p = 0.029); this was the only parameter that significantly differed between baseline and the recovery phase.

The average change observed in VO_2_ during PCT was 14.43 (95% CI -27.28 to 56.14) ml/min, and during SOEOB was 90.69 ml/min (95% CI 44.04 to 137.34), demonstrating a statistically significant difference (p = 0.007) between the 2 activities (see Table 2). MV change scores were also significantly different, observed at 0.74 l/min (95% CI -0.92 to 1.56) during PCT and 2.86 l/min (95% CI 1.70 to 3.99) during SOEOB (p = 0.012). VCO_2_ change scores were not significantly different (p = 0.051), but did show a change of 11.05 ml/min (95% CI -14.58 to 36.68) during PCT compared to 34.31 ml/min (95% CI 5.20 to 63.41) during SOEOB. No significant differences were observed in the change scores of MAP or HR when comparing PCT and SOEOB. MAP showed a change of 9.37 mmHg (95% CI 4.35 to 14.39) during PCT and 8.78 mmHg (95% CI 0.84 to 16.71) during SOEOB (p = 0.575); HR increased by 8.08 bpm (95% CI 2.75 to 13.41) and 7.31 bpm (95% CI 2.59 to 12.03) during PCT and SOEOB respectively (p = 0.705).

**Table 2:** **Baseline to activity change scores presented as mean ± SD**

Parameter	Baseline to activity change scores		p value
	PCT	SOEOB	
VO_2_ (ml/min)	14.43 *(-27.28 to 56.14)*	90.69 *(44.04 to 137.34)*	p = 0.007*
VCO_2_ (ml/min)	11.05 *(-14.58 to 36.68)*	34.31 *(5.20 to 63.41)*	p = 0.051
MV (l/min)	0.74 *(-0.92 to 1.56)*	2.86 *(1.70 to 3.99)*	p = 0.012*
MAP (mmHg)	9.37 *(4.35 to 14.39)*	8.78 *(0.84 to 16.71)*	p = 0.575
R (bpm)	8.08 *(2.75 to 13.41)*	7.31 *(2.59 to 12.03)*	p = 0.705

*Indicates p value < 0.05.

Two adverse events were observed during the study, both occurred during the activity of SOEOB, one participant desaturated due to condensation collecting in the ventilation tubing, the other experienced an increase in HR beyond the pre-set 80% maximal HR threshold. In both cases the intervention was terminated and the adverse effects stabilised immediately.

VCO_2_ values during activity PCT were missing for one participant due to equipment error. The occurrence of missing data in the other 9 patients was less than 5%, and in these circumstances the last value was carried forward [21]. The majority of missing data occurred when the participant did not have an arterial line in situ and BP readings were taken non-invasively; for practical reasons, it proved difficult to take readings minute-by-minute. The remainder occurred due to equipment error in obtaining values for VO_2_ and VCO_2_.

## Discussion

The main finding of our study was that SOEOB was associated with a significant increase in VO_2_ in comparison to PCT. During SOEOB, significant increases were seen in all parameters measured, before returning to baseline during the recovery phase. PCT elicited a minimal increase in VO_2_, VCO_2_ and MV suggesting a low metabolic demand, but, perhaps interestingly this was associated with a significant increase in MAP and HR, with HR remaining elevated even into the recovery phase.

SOEOB has been shown to elicit a cardiorespiratory response in the critically ill [16, 17], but few studies have investigated the effects of passive exercise and there has been no previous comparison between active and passive sitting. SOEOB has been more widely investigated in rehabilitation programs demonstrating progressive increases in cardiorespiratory parameters. Zafiropoulos *et al*. [16] only found significant increases in MAP between supine to active sitting, however patients were taken off mechanical ventilation before mobilisation, suggesting low dependency within the patient sample investigated. Stiller *et al*. [17] studied a more dependent patient sample, finding significant increases in HR and BP during SOEOB; however they did not measure metabolic or respiratory parameters. In the current study, the increase in VO_2_ and VCO_2_ observed during SOEOB, may be explained by greater muscle activity during this task with subsequent increase in energy requirements.

PCT did not elicit any significant changes in respiratory parameters in comparison to resting supine, results that concur with those found in a recent similar study [14]. This study also reported a significant increase in HR beyond activity completion. The authors concluded that the changes were of minor clinical significance as, similarly to this study, any increases were of the magnitude less than 10%. Our results suggest no increase in metabolic demand with PCT; perhaps the observed cardiovascular changes relate to an orthostatic response to upright positioning, a hypothesis which may warrant further investigation.

Despite a growing interest in rehabilitation of the critically ill patient [22], there remains limited data describing the metabolic cost of exercise within the critically ill; with few studies reporting VO_2_ as a measure of exercise response. The protocol used in this study, was previously used to investigate the impact of chest physiotherapy on VO_2_ [23]; the authors describe transient increases in VO_2_ during tasks requiring increased muscular activity such as turning and coughing. Jones and Dean [24] also found that positions with a reduced base of support, and subsequent increase in postural muscle activity were associated with a higher VO_2_. The authors suggest that VO_2_ reflects the metabolic response to physiological stress and measurement of VO_2_ during rehabilitation may allow physiotherapists to quantify the increased muscular oxygen demand. This may help identify patients’ ability to tolerate exercise and help direct prescribed activity.

In our study, the protocol was interrupted once for desaturation to 88% and once for tachycardia beyond the 80% maximal HR threshold. These instances may reflect the limited reserve of the individual patients; however, both were resolved without any detriment to the patients concerned. In a systematic review of early mobilisation in intensive care, desaturation is the most commonly reported event; serious adverse events are rare [25]. Stiller *et al.* [17] surmised that a reduction in SpO_2_ indicates that patients do not have the cardiorespiratory reserve to meet the additional demands of exercise. In an earlier review, the same authors conclude the potential benefits mean rehabilitation even in the presence of marginal cardiorespiratory reserve is important, but recommend monitoring and re-assessment during rehabilitation sessions [15].

This study has several limitations. The primary outcome measure of VO_2_ was determined by indirect calorimetry which is very sensitive to movement and change in breathing pattern, both of which occur during exercise [26]. However, this study was pragmatic in nature and as these measurements are easily accessible in clinical practice, VO_2_ was deemed an appropriate outcome measure. The cross-over design reduces the impact of differences seen in the baseline characteristics, but we recognise the sample size was small and equipment failure did mean some data was missing, which may affect the validity of the results.

Exercise prescription in the critically ill is complex, partly due to the unstable nature of these patients, and also due to the lack of standardised outcome measures to assess the effect of interventions provided [27]. We have demonstrated the metabolic demands of early exercise activities commonly used in critical care physiotherapy practice; the same method could now be employed to evaluate further progressive exercise activities, informing exercise prescription in this setting. Severity or chronicity of critical illness was not taken into account, and may have had a real impact on the elicited exercise response. Further work is required examining the differences in the exercise response between acute and chronic critical illness and to evaluate any impact on the rehabilitation prescription in these different patient populations.

## Conclusions

We conclude that SOEOB is a more metabolically demanding activity than a PCT in stable, mechanically ventilated, critically ill patients. This likely reflects the increased demand of the muscles in order to maintain active sitting and may help tailor rehabilitation prescriptions for the individual critically ill patient. During critical illness, demand for oxygen increases, in addition to compromised oxygen delivery. As such, initiating rehabilitation and determining the degree of exercise intensity are important decisions for clinicians; the results of this study add to our understanding of the exercise response in critically ill patients. Further studies are needed to investigate the metabolic demands of other exercise activities routinely employed in the intensive care setting and to determine how the stage of critical illness has an impact on the exercise response observed in this patient population.

## Authors’ information

NC wrote this manuscript as part of a dissertation towards an MSc awarded by Sheffield Hallam University, while working at University Hospital Southampton. RC is a consultant in critical care at University Hospital Southampton and supported the study during its completion.

## Abbreviations

SOEOB: Sitting on the edge of the bed
PCT: Passive chair transfer
VO_2_: Oxygen consumption
VCO_2_: Carbon dioxide consumption
MV: Minute ventilation
MAP: Mean arterial pressure
HR: Heart rate
ECG: Electrocardiogram
SBP: Systolic blood pressure
S_p_O_2_: Pulse oximetry
PEEP: Positive end-expiratory pressure
Hb: Haemoglobin
DVT: Deep vein thrombosis
PE: Pulmonary embolism
EVV: Engström Elvira ventilator
RR: Respiratory rate
TV: Tidal volume.
